# Experimental designs for phase I and phase I/II dose-finding studies

**DOI:** 10.1038/sj.bjc.6602969

**Published:** 2006-01-24

**Authors:** J O'quigley, S Zohar

**Affiliations:** 1 Institut Curie, Paris, France; 2 Centre d'Investigation Clinique, INSERM, Hôpital Saint Louis, Paris, France; 3 U717 INSERM, Hôpital Saint Louis, Paris, France

**Keywords:** clinical trial, continual reassessment method, dose escalation, dose-finding studies, maximum tolerated dose, phase1 trial, toxicity

## Abstract

We review the rationale behind the statistical design of dose-finding studies as used in phase I and phase I/II clinical trials. We underline what the objectives of such dose-finding studies should be and why the widely used standard design fails to meet any of these objectives. The standard design is a ‘memoryless’ design and we discuss how this impacts on practical behaviour. Designs introduced over the last two decades can be viewed as designs with memory and we discuss how these designs are superior to memoryless designs. By superior we mean that they require less patients overall, less patients to attain the maximum tolerated dose (MTD), and concentrate a higher percentage of patients at and near to the MTD. We reanalyse some recently published studies in order to provide support to our contention that markedly better results could have been achieved had a design with memory been used instead of a memoryless design.

There is an average of 10 years between the development of improved statistical methods and their implementation in practice. In the area of phase I dose-finding studies, this average is exceeded since a review of the literature indicates that most current studies are being carried out according to statistical designs, which are over half a century old. Furthermore, it had been demonstrated that these old designs are inefficient and inferior to new designs published in the literature since the late nineteen eighties.

The old (although still currently widely used) designs can be described as memoryless designs as opposed to the majority of the new ones, which can be labelled as designs with memory. In the following sections, we clarify just what we mean by the term ‘memoryless’. Following this we describe designs with memory and the advantages, which follow from such a property. Finally, we consider several recently published studies and indicate how we could have performed better had more efficient designs been used. Firstly, we recall the basic principles of phase I dose-finding studies, in particular in the context of cytotoxic agents.

## BACKGROUND

**Figure 1:**
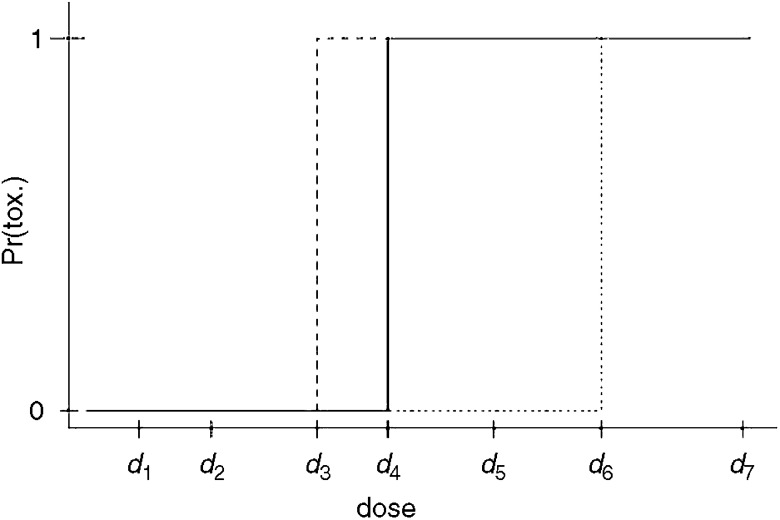
Dose toxicity curve for three patients.

**Figure 2:**
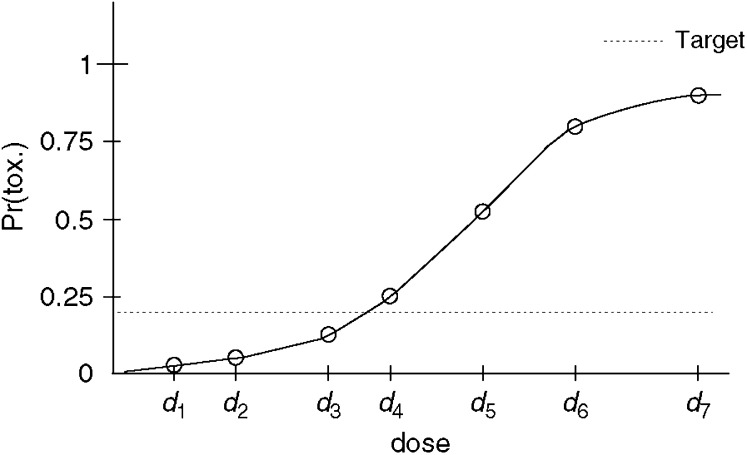
Dose toxicity curve for a hypothetical population of patients.

For cytotoxic anticancer drugs, it is assumed that there exists a dose–toxicity effect whereby the higher the dose, the greater the risk of observing dose-limiting toxicity. The goal of dose-finding studies is to find the highest tolerable dosage: the maximum tolerated dose (MTD) that corresponds to some given acceptable toxicity rate. In studies of treatment efficacy, similar methodology is employed aiming to identify a dose capable of producing a given rate of success. A model that is widely assumed for cytotoxics, although (as for any model) open to debate, is a model that assumes monotonicity. This means that when a patient experienced a dose-limiting toxicity at a specific level, then, had this same patient been treated at any higher level he would also have suffered dose-limiting toxicity. Conversely, were the patient to tolerate the treatment at a specific dosage, then, for all lower levels, the patient would also have tolerated treatment. This is reasonable in most cases but might need to be questioned in certain situations, for example, for immunological therapies. Figure 1 illustrates three such dose toxicity curves for three hypothetical patients. Owing to patient variability, each patient responds at a different level. Some patients are able to tolerate higher levels of treatment than others. Most often we will not be able to know for any individual just what their particular dose toxicity (0, 1) step function might be; in other words, the lowest level at which the patient would encounter a dose-limiting toxicity. However, we can learn this for a group of subjects. In the light of patient variability, the lowest level for one patient may differ from that for another. Were we to take an average over the three patients, we would have a step function with steps of size 1/3 at the observed change points. Were we to target a level where 33% of subjects encounter a DLT, then we could estimate a point above dose *d*_3_ and below dose *d*_4_. This would of course be a rough estimate, since three patients may not be enough to capture the effects of a much larger group. However, as we include bigger samples, we can conceive of the simple step function for three patients becoming more refined and eventually looking something like Figure 2. In practice, we will not know such a curve and the problem is to find the dose *d*_*i*_ such that some given percentage of patients will encounter a DLT at this dose and higher doses. Technically, our problem is to inverse the curve in Figure 2. This inversion would tell us which dose corresponds to some given rate of toxicity.

### Experimental and ethical constraints on phase I studies of cytotoxics

The precise goals of a phase I dose-finding study are not always clearly defined. The absence of such definitions and the lack of clinically motivated exigencies have led to the use of a number of schemes, in particular the standard design, having properties that are clearly undesirable. We call such designs ‘memoryless’ and we describe them in the next section. It was argued (O'Quigley *et al*, 1990) that the clinical requirements of a phase I dose-finding study should be to the following:

1. minimize the number of under-treated patients, that is, patients treated at unacceptably low-dose levels;
2. minimize the number of overtreated patients, that is, patients treated at unacceptably high-dose levels;
3. minimize the number of patients needed to complete the study (efficiency); and
4. respond quickly to inevitable errors in initial guesses, rapidly escalating in the absence of indication of drug activity (toxicity) and rapidly de-escalating in the presence of unacceptably high levels of observed toxicity.

The reason argued that we need to consider accepting toxicity in phase I cancer studies is because, without some toxicity, we will not see any efficacy and *vice versa*. Indeed, it is common in this context to view toxicity as a surrogate for efficacy. The question then is how to control for the amount of toxicity. In the light of these observations, the goal of a phase I study of cytotoxics is to identify a target dose level at which the rate of toxicity is as close as possible to some predetermined rate, typically 20–30%. The goal is to identify this level at the end of the study but also, during the study itself, to concentrate as many patients as possible at and around this target level. Designs with memory achieve this goal. Memoryless designs do not.

## MEMORYLESS DESIGNS

The standard design is a memoryless design. This includes three patients at the lowest dose level. If all three patients tolerate the dose, then a further three patients are included at the dose immediately above the lowest level. This is continued until a DLT is encountered. If there are two or more of these in the group of three patients then the dose is considered unacceptable and a further three patients are included at the level just below this. If no more than one toxicity is encountered among that group of three patients, then this level is recommended as the MTD. Otherwise, we descend a further level until this is the case, in which case the level is then defined as the MTD.

The standard design is a so-called random walk that incorporates a stopping rule. The stopping rule brings the study to a halt as soon as two or more toxicities have been observed at a level and, for all levels below this, we define the MTD as the highest level at which we observe no more than one toxicity out of a total of six patients. There are a number of variants upon this scheme, but they all share an essential property; allocation to the next dose level for an incoming group of three patients only depends upon what has happened to the total of six patients treated at that level. All other information concerning other dose levels and the distribution of toxicities are ignored. Thus the information is lost. These designs are in consequence memoryless.

This is a serious shortcoming and has been pointed out by several authors (Faries, 1994; Korn *et al*, 1994; Goodman *et al*, 1995). In more involved situations, things get worse. Take, for instance, the two group case, a case that arises frequently, possibly in the majority of studies. A common example arises when we have one group defined as having received relatively heavy prior treatment and a second group having relatively less prior treatment. We anticipate the first of these groups to have an MTD no greater than that for the second group. Yet, for the standard design, there is no way to use this information. Parallel studies need to be run and what happens in one group has no impact on treatment allocation for the other.

## DESIGNS WITH MEMORY

The main idea to designs with memory leans on a fundamental statistical principle -all the information should be used. As the study progresses and new patients are included, then our estimate of the MTD becomes more precise. The most well-known design with memory is the continual reassessment method (CRM) (O'Quigley *et al*, 1990). The basic idea is to directly address the first two requirements for phase I studies, outlined above; (1) we should minimize the number of undertreated patients, that is, patients treated at unacceptably low-dose levels and (2) we should minimize the number of patients treated at unacceptably high-dose levels. The MTD can then be viewed as the level such that, above it we are overtreating, below it we are undertreating. We would aim to treat as many patients as possible at the MTD. The essential nature of the CRM (Gasparini and Eisele, 2000) is:

1. an allocation rule to assign sequentially the incoming patients to one of the possible doses, with the intent of assigning doses ever closer to, and eventually recommending, the MTD;
2. a statistical procedure that updates the information on the probabilities of toxicity in light of the results obtained for the patients already observed.

Apart from the dose levels we can assume, for the simplest designs the only observation we will make will be whether or not we observed a DLT. A DLT can indicate: (1) the level is high enough for producing undesirable side effects, (2) viewing a DLT as a surrogate for efficacy; the level is high enough for producing some treatment effect. Of course we do not have two types of DLT, toxicity DLT and an efficacy DLT. We only have one and this leads us to consider some rate of observation of DLT as being satisfactory. We consider that there exists some level, among those available, producing a rate of DLTs such that significantly higher rates would be viewed as being too toxic, and significantly lower rates would be viewed as producing insufficient response.

The level producing this satisfactory rate is called the target level, or the MTD. There is some arbitrariness in the choice of this rate, and input from the clinicians is needed. The older standard design was based largely upon the idea of escalating to a level where we observe, on average, one DLT in three patients, a level considered too toxic, so that the MTD was taken to be the level just below this, resulting in one DLT in four patients or one DLT in five patients. These rates 0.33, 0.25 and 0.2 are those most commonly used in phase I studies. Apart from practical considerations, nothing prevents the investigator from working with other rates.

Once the investigators have decided upon an acceptable target rate, the MTD is then defined to be the level producing a rate of toxicity as close as possible to this rate. The aim of the continual reassessment method is not only to identify such a level but, in the light of all available information, to treat each included patient at our best guess of this level. Each patient adds to this information, hence the term ‘reassessment’. The important idea is that of updating whatever information we have after each observation or group of observations.

### Worked illustration

In order to be able to treat each included patient at the level producing a rate of DLTs closest to some, acceptable, target rate. which we denote as *θ*, we need estimates of the DLT rate at each dose. There were six dose levels, denoted *d*_1_ to *d*_6_. The unknown probability of toxicity of observing a DLT at dose level *d*_*i*_ is denoted by *R*(*d*_*i*_), for *i* taking values from 1 to 6. Our problem is to estimate *R*(*d*_1_) to *R*(*d*_6_) in the light of observations made. We will use a simple working model, to carry out this estimation. Our working model is chosen such that our estimates *R̂*(*d*_*i*_) of the unknown *R*(*d*_1_)<*R*(*d*_2_) << *R*(*d*_6_) and *R*(*d*_1_)<*R*(*d*_2_)<…<*R*(*d*_6_) as well as *R̂*(*d*_1_)<*R̂*(*d*_2_) << *R̂*(*d*_6_). In this computer illustration, the true toxic probabilities were *R*(*d*_1_)=0.03, *R*(*d*_2_)=0.22, *R*(*d*_3_)=0.45, *R*(*d*_4_)=0.60, *R*(*d*_5_)=0.80 and *R*(*d*_6_)=0.95, these probabilities being unknown. The target toxicity rate was chosen to be *θ*=0.20 (O'Quigley and Shen, 1996). The MTD is then dose level 2 where the true toxicity probability is 0.22. As an aside, the most effective CRM designs are so called two-stage designs; an early escalation stage followed by a CRM guided modelling stage. Following an initial escalation strategy mimicking the standard design, we have the following observations on nine patients;

- level 1: 0 toxicities/three patients
- level 2: 0 toxicities/three patients
- level 3: 2 toxicities and one non-toxicity/three patients.

On the basis of our model we have, *R̂*(*d*_1_)=0.101, *R̂*(*d*_2_)=0.149, *R̂*(*d*_3_)=0.316, *R̂*(*d*_4_)=0.472, *R̂*(*d*_5_)=0.652 and *R̂*(*d*_6_)=0.775. The 10th entered patient is then treated at level 2 for which *R̂*(*d*_2_)=0.149 since, from the available estimates, this is the closest to the target *θ*=0.20. The 10th included patient does not suffer toxic effects and, as a result, all our probability estimates are revised downward, only very slightly since such an observation does not contain much information. We now estimate the six probabilities of toxicity as;

*R̂*(*d*_1_)=0.070, *R̂*(*d*_2_)=0.133, *R̂*(*d*_3_)=0.295, *R̂*(*d*_4_)=0.451, *R̂*(*d*_5_)=0.635, and *R̂*(*d*_6_)=0.763. Once again the level that turns out to have an estimated probability of toxicity the closest to *θ*=0.20 is level 2. Thus the 11th included patient is treated at level 2. Continuing in this way, it turns out that this same level, level 2, is in fact recommended to all of the remaining patients. After the inclusion of 16 patients, the recommended MTD is then level 2. The estimated probability of toxicity at this level is 0.212 and a 90% confidence interval for this probability is estimated as (0.07, 0.39).

## MEMORYLESS DESIGNS *VS* DESIGNS WITH MEMORY

The main perceived advantages of the standard design are threefold; its simplicity, its conservativeness in terms of cautious escalation and its ability to provide a reliable recommendation by using few patients. The first of these is true. The other two are not. These latter perceived advantages require closer scrutiny. Although it is very possible for the standard design to come to a conclusion by using few patients, there is a high cost, which accompanies this in that, corresponding to the small number of patients, the probability of an incorrect recommendation is unacceptably high (Reiner *et al*, 1999; Levy *et al*, 2001). Via simulations, those authors demonstrated that across a broad class of plausible situations, should the trial terminate after less than 16 patients, then the probability of correctly identifying the MTD would rarely exceed 20%, a performance that can only be described as lamentable.

## SOME RECENTLY PUBLISHED STUDIES REVISITED

Simulations, under a very wide array of possible situations, show that designs with memory, in particular the continual reassessment method, do better than memoryless designs. By ‘better’ we mean that they reach the MTD more quickly and that they treat more patients at and close to the MTD. The hypothetical aspect to simulations, although convincing enough for statisticians familiar with such tools, will sometimes leave the clinician sceptical. For a trial completed using a standard design, the clinician will often ask: what would the CRM or some other design with memory have performed had it been employed. It is not possible to provide an unequivocal answer to this question since, had a dynamic design with memory been used, the distribution of visited dose levels would generally change and we cannot know for certain what the responses at these levels would have been. However, following O'Quigley (2005), we are in a position to reanalyse completed real studies and, although we can not provide a fully determined answer to the question, we can provide useful estimates of the MTD that would have been identified by the competing design (given the data at hand and not simulated data) as well as dose levels visited during the study. While not giving an unqualified answer to the question (as no such answer exists), a retrospective analysis does throw further light on how we might expect to have performed had a design with memory been used in a number of recent studies.

### The lurtotecan trial

In this study, six different dose levels of lurtotecan were studied, namely, 1.5, 2.2, 3.3, 3.7, 4.0 and 4.9 mg m^−2^ (Giles *et al*, 2004). A total of 20 patients were included in the trial of which two (10%) patients were at dose level 1, 2 patients (10%) at dose level 2, 2 (10%) patients at dose level 3, 6 (30%) patients at dose level 4, 6 (30%) patients at dose level 5 and 2 (10%) patients at dose level 6. Table 1 represents the administered dose levels and the observed DLTs. At the end of the trial, the estimated MTD was 3.7 mg m^−2^. We applied a retrospective analysis on those data using the CRM (O'Quigley, 2005). The MTD is identified as level 4 using both the standard design and the CRM. The important observation to make is that the CRM would have included nearly 50% of the patients at the MTD in contrast to the 30% included at this level by the standard design. And this would have been a yet poorer figure had the investigators respected the protocol of 3 × 3 inclusions. As can be seen from the table, the early inclusions proceeded in groups of 2. Another important observation to be made is the percentage treated far from the MTD, either above or below. By far from the MTD, we mean by more than one level away from the MTD. 10% of patients were treated at level 6 using the standard design as opposed to 2% using the CRM. Such an observation confirms the simulation findings of earlier work (Korn *et al*, 1994). Of the patients, 20% were treated at levels 1 and 2 using the standard design as opposed to the figure of 15% using the CRM.

### The AMD473 and docetaxel trial

In this study 4 different dose levels of AMD473 and docetaxel were studied, namely, 80/60, 80/75, 100/75 and 120/75 mg m^−2^ of AMD473 and mg m^−2^ of docetaxel (Gelmon *et al*, 2004). A total of 33 patients were included in which eight (24%) patients at dose level 1, six patients (18%) at dose level 2, nine (27%) patients at dose level 3 and 10 (30%) patients at dose level 4 (Table 1). At the end of the trial the estimated MTD was 120/75 mg m^−2^. On applying a retrospective analysis on those data, the recommended dose level, once again, turns out to be the same. As for the preceding example, the allocation probabilities were considerably better under the CRM than the standard design. A total of 53% of patients would have received the MTD as opposed to the 30% under the standard design.

### The topotecan trial

A phase I dose finding study aiming to identify the MTD of intraperitoneal topotecan in combination with intravenous carplatin and paclitaxel in advanced ovarian cancer was described (Bos *et al*, 2005). Four different dose levels of topotecan were studied, 10, 15, 20 and 25 mg m^−2^. In this trial, 21 patients were included in which three (14%) patients at dose level 1, seven patients (33%) at dose level 2, six (28%) patients at dose level 3 and five (24%) patients at dose level 4 (Table 1). At the end of the trial, the estimated MTD was 20 mg m^−2^. Applying a retrospective analysis to these data, we found the recommended dose level to be again 20 mg m^−2^. From our simulation study, if a CRM design had been used, the MTD would have been administered to 51% of the patients, which is substantially better than the 28% receiving the MTD under the standard design, which was actually used.

### The amrubicin trial

Okamoto *et al* (2006) describe a phase I dose-finding trial of amrubicin in patients with refractory or relapsed lung cancer. Three different dose levels of amrubicin were studied, namely, 30, 35 and 40 mg m^−2^. In this trial 15 patients were included in which six (40%) patients at dose level 1, six patients (40%) at dose level 2 and three (20%) patients at dose level 3 (Table 1). At the end of the trial, the recommended dose was 35 mg m^−2^. On applying a retrospective analysis on those data, the recommended dose level was once again the same, that is, 35 mg m^−2^ a dose to which, we would have administered the MTD in 60% of the patients unlike the 40% obtained using the standard analysis.

## TOWARD MORE EFFICIENT AND MORE ETHICAL DESIGNS

The most ethical design possible would treat each patient at his or her own specific MTD. Were we in a position to do that, of course, no actual trial would be necessary. It is a lack of more precise knowledge that requires us to carry out the dose finding study. Even so, such an objective, even if wholly idealized and theoretical, can shed light on our endeavour. Specifically, as our knowledge improves, and it will do so in the course of any study, then such knowledge should be used efficiently to deliver a more accurate dose to the patient. Only the kind of models employed by designs with memory can achieve this. As an example, we can use prognostic information such as the degree of prior treatment to obtain a more accurate dose allocation for each patient (Geoerger *et al*, 2005). These ideas extend almost immediately and enable us to include in a single study patients with different prognoses, such as children and adults. Pursuing the goal of better adapting the treatment to the specifics relating to each individual patient, we can use pk/pd information (Piantadosi *et al*, 1998). Finally, as the clinical situation of these studies evolves and we make observations not just about toxicities, but also on measures of responses, then we can target quantities other than the MTD, in particular the MSD (the most successful dose), which combines information on toxicities and responses together. Only designs with memory can underwrite such studies and we believe that memoryless designs should be gradually phased out of use in the context of phase I and phase I/II dose-finding studies.

## Figures and Tables

**Table 1:** Dose-escalation and retrospective analysis

*The lurtotecan trial*						
Dose (mg m^−2^)	1.5	2.2	3.3	3.7	4.0	4.9
No. of patients	2	2	2	6	6	2
No. of patients with DLT	0	0	0	2	3	2
Estimated probabilities of toxicity from the data	0	0	0	0.33	0.5	1
Relative frequencies of allocation	0.1	0.1	0.1	0.3	0.3	0.1
*Retrospective analysis*						
Mean relative frequencies of allocation using CRM	0.05	0.1	0.13	0.49	0.21	0.02
Recommended dose level using retrospective CRM				X		
*The AMD473 and docetaxel trial*						
Dose (mg m^−2^–mg m^−2^)	80/60	80/75	100/75	120/75		
No. of patients	8	6	9	10		
No. of patients with DLT	1	1	2	3		
Estimated probabilities of toxicity from the data	0.125	0.17	0.22	0.30		
Relative frequencies of allocation	0.24	0.18	0.27	0.30		
*Retrospective analysis*						
Mean relative frequencies of allocation using CRM	0.07	0.16	0.22	0.53		
Recommended dose level using retrospective CRM				X		
*The topotecan trial*						
Dose(mg m^−2^)	10	15	20	25		
No. of patients	3	7	6	5		
No. of patients with DLT	0	1	1	3		
Estimated probabilities of toxicity from the data	0	0.14	0.17	0.6		
Relative frequencies of allocation	0.14	0.33	0.28	0.24		
*Retrospective analysis*						
Mean relative frequencies of allocation using CRM	0.08	0.22	0.51	0.19		
Recommended dose level using retrospective CRM			X			
*The amrubicin trial*						
Dose (mg m^−2^)	30	35	40			
No. of patients	6	6	3			
No. of patients with DLT	1	2	3			
Estimated probabilities of toxicity from the data	0.17	0.33	1			
Relative frequencies of allocation	0.4	0.4	0.2			
*Retrospective analysis*						
Mean relative frequencies of allocation using CRM	0.33	0.6	0.07			
Recommended dose level using retrospective CRM		X				

X=the recommended dose level.
